# Characterization of two relacidines belonging to a novel class of circular lipopeptides that act against Gram‐negative bacterial pathogens

**DOI:** 10.1111/1462-2920.15145

**Published:** 2020-07-20

**Authors:** Zhibo Li, Parichita Chakraborty, Reinder H. de Vries, Chunxu Song, Xinghong Zhao, Gerard Roelfes, Dirk‐Jan Scheffers, Oscar P. Kuipers

**Affiliations:** ^1^ Department of Molecular Genetics University of Groningen Groningen the Netherlands; ^2^ Department of Molecular Microbiology University of Groningen Groningen the Netherlands; ^3^ Stratingh Institute for Chemistry University of Groningen Groningen the Netherlands; ^4^ College of Resources and Environmental Science, National Academy of Agriculture Green Development, Key Laboratory of Plant‐Soil Interaction Ministry of Education, China Agricultural University Beijing 100193 China

## Abstract

The development of sustainable agriculture and the increasing antibiotic resistance of human pathogens call for novel antimicrobial compounds. Here, we describe the extraction and characterization of a class of cationic circular lipopeptides, for which we propose the name relacidines, from the soil bacterium *Brevibacillus laterosporus* MG64. Relacidines are composed of a fatty acid side chain (4‐methylhexanoic acid) and 13 amino acid residues. A lactone ring is formed by the last five amino acid residues and three positively charged ornithines are located in the linear fragment. Relacidines selectively combat Gram‐negative pathogens, including phytopathogens and human pathogens. Further investigation of the mode of action revealed that relacidine B binds to the lipopolysaccharides but does not form pores in the cell membrane. We also provide proof to show that relacidine B does not affect the biosynthesis of the cell wall and RNA. Instead, it affects the oxidative phosphorylation process of cells and diminishes the biosynthesis of ATP. Transcription of relacidines is induced by plant pathogens, which strengthens the potential of *B. laterosporus* MG64 to be used as a biocontrol agent. Thus, we identified a new group of potent antibiotic compounds for combating Gram‐negative pathogens of plants or animals.

## Introduction

Antimicrobials are substances that kill or inhibit the growth of microorganisms. They are of great value in different applications. Some of them are being used as weapons for plant disease biocontrol (Ongena and Jacques, 2008), while others are used as antibiotics for preventing and curing bacterial infections in animals, including humans (Chandra and Kumar, 2017). The discovery of novel antimicrobials is of paramount importance not only for the development of sustainable agriculture but also to overcome the antibiotic resistance, which has become one of the biggest threats to human health in recent decades.

Soil is an ecosystem that hosts a large and diverse population of microorganisms (Roesch *et al*., 2007). Microbes develop strategies to adapt to fluctuating soil environments and to survive in the competition with other organisms. Production of antimicrobials is one of the most potent strategies for this adaptation (Davies, 1990). Thus, soil microorganisms form a natural reservoir of antimicrobials (Chandra and Kumar, 2017). Screening of soil microorganisms that harbour novel biosynthetic gene clusters (BGCs) against pathogens is a traditional but efficient way to discover novel antimicrobials.

In a previous study, we isolated a rhizosphere bacterium, *Brevibacillus laterosporus* MG64, which displayed potent activity against plant pathogens and mammalian pathogens (Li *et al*., 2020). *Brevibacillus* is a genus of bacteria reclassified from *Bacillus* based on the 16S rRNA sequence analysis (Shida *et al*., 1996). It is a rich resource for antimicrobials and many compounds have been isolated and characterized (Yang and Yousef, 2018). For instance, gramicidin S, loloatins, and tyrocidines were discovered from *Brevibacillus brevis* (Hotchkiss and Dubos, 1941; Gause and Brazhnikova, 1944; Gerard *et al*., 1999), while tauramamide, bogorols, laterosporulin, and so on were isolated from *B. laterosporus* (Todd Barsby, 2001; Barsby *et al*., 2006; Desjardine *et al*., 2007; Singh *et al*., 2012). The current study was initiated to unveil novel antimicrobials produced by *B. laterosporus* MG64, which harbours abundant novel BGCs (Li *et al*., 2020). The bioactivity against different kinds of pathogens and the underlying mode of actions of the bioactive compounds were further investigated and dissected in order to evaluate their potential in applications.

## Results

### Purification and identification of relacidines

We previously isolated *B. laterosporus* MG64 from an agricultural rhizosphere sample and showed that this organism has several biosynthetic gene clusters encoding potential antimicrobials and secretes compounds that inhibit the growth of bacterial and fungal pathogens (Li *et al*., 2020). To characterize these antimicrobials, supernatant from a *B*. *laterosporus* MG64 culture was precipitated using ammonium sulfate and applied to HPLC for purification. Each peak that eluted from the HPLC was collected for an *in vitro* activity test using *X*. *campestris* pv. *campestris* as an indicator. Two peaks (Supporting Information Fig. S1) that displayed the most potent activity against the indicator were subjected to LC–MS to determine their molecular masses. The first peak showed *m/z* values of 1549.82 and 1571.80, corresponding to a singly protonated compound [M + H]^+^ and its sodium‐cationized ion [M + Na]^+^ respectively. The second peak showed *m/z* values of [M + H]^+^ 1563.83 and [M + Na]^+^ 1585.82 (Supporting Information Fig. S2). The similar elution time and molecular mass suggested that they belong to the same class of compounds. We designated them as relacidine A and relacidine B respectively. Relacidines represent some of the major secondary metabolites produced by *B. laterosporus* MG64. With the method used, around 0.2 mg of relacidine A and 0.5 mg of relacidine B can be obtained from 1 l of culture.

**Fig 1:**
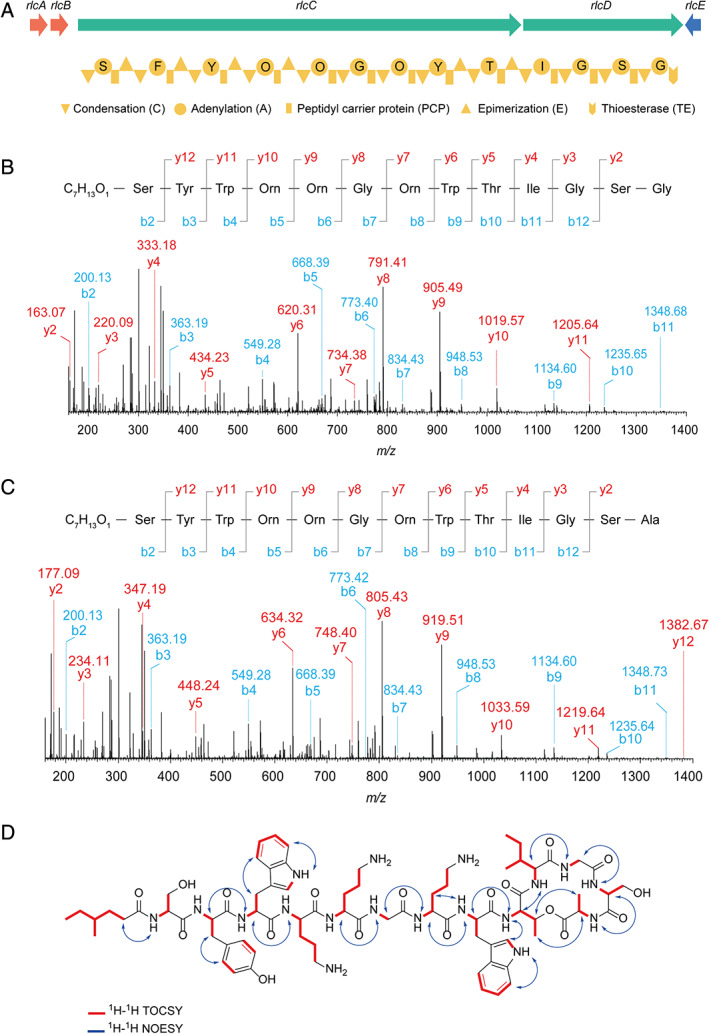
Characterization of biosynthetic gene cluster and structure of relacidines. A. Gene cluster of relacidines predicted by antiSMASH. ABC transporter genes are indicated in red; core biosynthetic genes are shown in green; drug resistance related gene is labelled with blue. The letters in the adenylation domains represent the predicted amino acid residues. Tandem MS analysis of hydrolyzed relacidine A (b) and relacidine B (c). Peptides were hydrolyzed with 2 M NaOH and desalted before applying to LC–MS/MS. (d) ^1^H‐^1^H TOCSY and ^1^H‐^1^H NOESY NMR crosspeaks of relacidine B.

To further characterize the compounds, the contents of both peaks were applied to LC–MS/MS analysis. As shown in the Supporting Information Fig. S3, both compounds were identified to contain a fragment of Tyr‐Trp‐Orn‐Orn‐Gly‐Orn‐Trp. With this information and the prediction by antiSMASH (Blin *et al*., 2019), we successfully identified the gene cluster of relacidines. This gene cluster contains five genes, including two ATP‐binding cassette transporter genes (*rlcA* and *rlcB*), two large core biosynthetic genes (*rlcC* and *rlcD*) and one drug resistance transporter gene (*rlcE*). The core biosynthetic genes encode 13 modules, among which the module 4 to module 7 incorporating the identified fragment of Orn‐Orn‐Gly‐Orn (Fig. 1A, Supporting Information Fig. S3). According to the prediction, the final product should contain 13 amino acid residues. However, the signals from the C terminus were largely missing in the LC–MS/MS data, suggesting the potential existence of a cyclic structure, which is prevalent in natural products. To confirm this hypothesis, each compound was hydrolyzed with 2 M NaOH, desalted, and subjected to LC–MS/MS analysis. The signals at the C termini were found after hydrolysis, indicating the presence of a ring in the original compounds (Fig. 1B and C). The ring of relacidine A was determined to be constituted of Thr‐Ile‐Gly‐Ser‐Gly, which is in perfect accordance with the prediction from antiSMASH (Fig. 1A). Relacidine B has the same *b* ions as relacidine A, but the *y* ions show a difference of 14 Da, which indicates their difference at the last two amino acid residues of the C terminal region (Fig. 1B and C). Residue Ser‐12 (the number indicates the position of the amino acid residue, the same for the rest below) was predicted with high confidence by antiSMASH, and therefore we speculate the last amino acid residue of relacidine B to be Ala. The fatty acid tail and the first amino acid residue are predicted to be C_7_H_13_O_1_ and Ser, respectively, which is supported by the tandem MS data (Fig. 1B and C).

The structure of relacidine B was further confirmed by 1D and 2D NMR spectroscopy, where ^1^H NMR, ^1^H‐^1^H‐TOCSY NMR, ^1^H‐^1^H‐COSY NMR, and ^13^C‐^1^H‐HSQC NMR techniques were used to identify the ^1^H and ^13^C signals of the different amino acid residues. All chemical shifts found were consistent with the MS/MS results. The structure of the fatty acid side chain was found to be 4‐methylhexanoic acid (Fig. 1D). Cross‐peaks in the ^1^H‐^1^H‐NOESY NMR verified the amino acid sequence found for this peptide in MS/MS (Fig. 1d). Moreover, NOESY cross‐peaks of both Ile10 and Ala‐13 with Thr‐9 confirmed the presence of the lactone macrocycle (Fig. 1D). The chemical shift assignments are summarized in the Supporting Information Table S1.

Taken together, relacidines are identified as novel lipopeptides that are constituted with a fatty acid tail (4‐methylhexanoic acid) and 13 amino acid residues. Moreover, the last amino acid residue was linked to Thr‐9 with an ester bond, thus forming a lactone ring (Fig. 1D). Relacidines contain three positively charged ornithine residues, resulting in a net positive charge at neutral pH conditions, and therefore are classified as cationic peptides, which are considered to be attractive therapeutic candidates to combat Gram‐negative pathogens (Hancock, 2001; Ntwasa, 2012).

### Antibacterial activity and mechanism of action

The relacidines were assessed for antibacterial activity. Phytopathogens (*Xanthomonas* species, *Pseudomonas syringae*, *Pectobacterium carotovorum*, and *Ralstonia syzygii*), food pathogen (*Bacillus cereus*), and human pathogens (*E. coli* ET8, *Klebsiella pneumoniae*, *Pseudomonas aeruginosa*, *Staphylococcus aureus*, and *Enterococcus faecium*) were tested in order to evaluate the potential of relacidines in different applications. As shown in Table 1, the relacidines displayed potent activity against all the Gram‐negative bacteria tested. The minimum inhibitory concentration (MIC) values are in the range of 0.25–2 μg ml^−1^ (0.16–1.29 μM), which is comparable to that of polymyxin B, a cationic peptide antibiotic commonly used to treat infections caused by multiple drug‐resistant pathogens (Velkov *et al*., 2013). The addition of exogenous lipopolysaccharides (LPS) from *E. coli* increases the MIC values of both relacidines and polymyxin B, suggesting their binding to LPS. Relacidines did not affect the growth of the tested Gram‐positive bacteria (i.e. *Staphylococcus aureus subsp. aureus* 533 R4, *Bacillus cereus* ATCC 14579, and *Enterococcus faecium* LMG16003) at a concentration up to 32 μg ml^−1^. A similar phenomenon was observed for polymyxin B, where activity against Gram‐positive bacteria was not observed.

**Table 1:** MIC values of relacidines against selected pathogenic bacteria.

Type of pathogen	Pathogen	MIC (μg mL^−1^)		
		Relacidine A	Relacidine B	Polymyxin B
Gram‐negative	*Xanthomonas campestris* pv. *campestris* NCCB92058	0.5	0.25–0.5	≤0.06
	*X. campestris* pv. *campestris* NCCB92058 + LPS (100 μg ml^−1^)	4	4	8
	*Xanthomonas translucens* pv. *graminis* LMG587	0.25	0.25	≤0.06
	*Pseudomonas syringae* pv. *antirrhin* LMG2131	0.5	0.5	0.12
	*Pseudomonas syringae* pv. *tomato* DC3000	0.5	0.5	0.12
	*Pectobacterium carotovorum* LMG5863	2	0.5	0.25
	*Ralstonia syzygii* subsp. *syzygii* LMG6969	2	1	0.25
	*Escherichia coli* TOP10	2	2	0.25
	*E. coli* TOP10 + LPS (100 μg ml^−1^)	8	8	8
	*E. coli* ET8	2	2	0.25
	*Klebsiella pneumoniae* LMG20218	2	2	0.25
	*Pseudomonas aeruginosa* PAO1	2	2	0.5
Gram‐positive	*Staphylococcus aureus subsp. aureus* 533 R4	>32	>32	>32
	*Bacillus cereus* ATCC 14579	>32	>32	>32
	*Enterococcus faecium* LMG16003	>32	>32	>32

**Fig 2:**
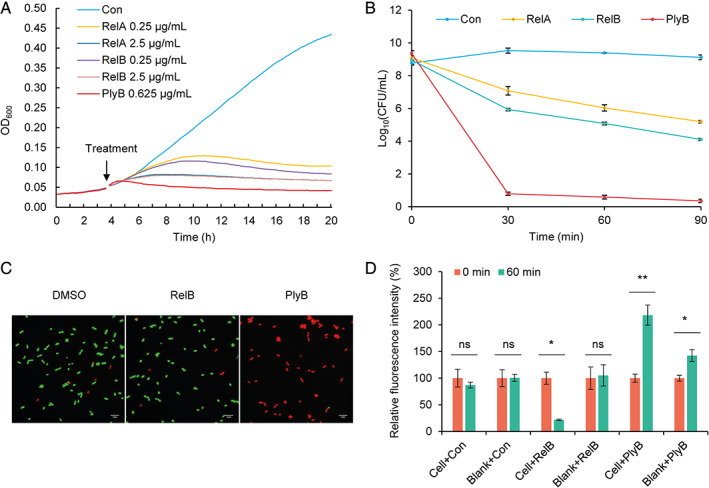
Growing curve, killing curve, membrane permeability, and membrane potential effect of relacidines. A. Growing curve of *X. campestris* pv. *campestris* exposed to relacidines. The overnight culture was diluted with fresh LB to an OD_600_ of 0.05 and dispensed into a 96‐well plate (100 μl each well). Compounds were added at different concentrations when the cells reach the early stationary phase. B. Killing curve of relacidines to *X. campestris* pv. *campestris*. All compounds were added at a concentration of 2.5 μg ml^−1^. C. Membrane permeability of relacidine B. *X. campestris* pv. *campestris* cells (OD_600_ = 0.2) were treated with different compounds (DMSO and 0.5 μg ml^−1^ relacidine B for up to 7 h, and 1 μg ml^−1^ polymyxin B for 5 min). Cells were washed with PBS buffer and stained with SYTO9 and PI before inspecting with a microscope. D. Membrane potential effect of relacidine B. The membrane potential dye DiSC(3)‐5 was added at a final concentration of 6 μM. Relacidine B and polymyxin B were added at final concentrations of 0.5 μg ml^−1^ and 1 μg ml^−1^ respectively. The fluorescence signal intensity is relative to the stabilized signal after adding DiSC(3)‐5 (0 min). Con, control (Milli‐Q water), RelB, relacidine B; PlyB, polymyxin B. ns, no significant difference; * significant difference (T‐test, *P* < 0.05), ** very significant difference (T‐test, *P* < 0.01).

The plant pathogen *X. campestris* pv. *campestris* was used to study the mode of action of relacidine B because of its great sensitivity. A growth curve was first determined to understand the efficacy of relacidines. Growth inhibition is defined as a drop in the increase of the optical density of a culture compared to the control. As shown in Fig. 2A, the control cells are continuously growing, while the cells treated with a relacidine or polymyxin B displayed slower growth after a defined period of time, followed by an absence of growth. The effective time of relacidines is around 2.5 h when applied at 1 × MIC and a faster effect was observed when the concentrations used are higher. Polymyxin B acts faster than relacidines when applied at the same MIC level. A time‐kill assay was conducted to determine whether relacidines have a bactericidal effect. The result showed that cell viability was significantly reduced after 30 min of exposure to relacidines at 10 times the MIC (Fig. 2B), which confirms the bactericidal effect of relacidines.

A common mechanism of action employed by a lot of antibiotics, including cationic peptides, is interfering with membrane integrity (Ntwasa, 2012). We analysed whether relacidine B can permeabilize the cell membranes of *X. campestris* pv. *campestris* using a combination of two fluorescent DNA dye, one (SYTO9) green and membrane permeable, and the other (propidium iodide) red and membrane impermeable. Cells treated with polymyxin B, a cationic peptide antibiotic known to disrupt cellular membranes (Khondker *et al*., 2019), were stained red, indicating that membranes were damaged. In contrast, cell membranes remain intact after treating with relacidine B and DMSO (control) for up to 7 h, indicating that relacidine B does not form holes in the cell membrane (Fig. 2C).

Next, we investigated the membrane potential of cells treated with relacidine B using the potential sensitive membrane dye DiSC(3)‐5 (Te Winkel *et al*., 2016). Considering that cationic peptides may affect the fluorescence signal, blanks without cells were also involved. As shown in Fig. 2D, the control treatment did not affect the signal regardless of the presence of cells. In contrast, the cationic peptide polymyxin B caused an increase in the blank, but the relative intensity of the signal is higher when cells were present. This is in accordance with its pore formation in the cell membrane. Relacidine B, which is identified to be a cationic peptide in this study, is compatible with the dye and the fluorescence signal was decreased when the cells were present. This suggests that the hyperpolarizing effect of relacidine B on cell membranes and further proved the integrity of cell membranes.

Inhibition of macromolecule (such as peptidoglycan, RNA) biosynthesis is another common mode of action employed by antibiotics. To investigate whether peptidoglycan biosynthesis is affected by relacidine B, incorporation of the fluorescent d‐amino acid analogue hydroxycoumarin‐carboxylic acid‐amino‐d‐alanine (HADA) was followed. The incorporation of HADA in the cells treated with relacidine B or DMSO (negative control) was not affected, while that in the cells exposed to ceftriaxone (positive control) was significantly reduced (Supporting Information Fig. S4). This result suggests that relacidine B does not abolish the biosynthesis of peptidoglycan. A competition experiment in which lipid II was added to relacidine B before treatment confirmed that the peptidoglycan precursor lipid II, which is the target for many membrane‐active peptides, is not the target for relacidine (Supporting Information Fig. S5). To investigate RNA synthesis during relacidine treatment, incorporation of [5^3^H] uridine in RNA was followed over time. As shown in the Supporting Information Fig. S6, rifampicin blocks incorporation of [5^3^H] uridine, whereas relacidine B had no effect and was comparable to the control. This result indicates that relacidine B does not affect the biosynthesis of RNA.

**Fig 3:**
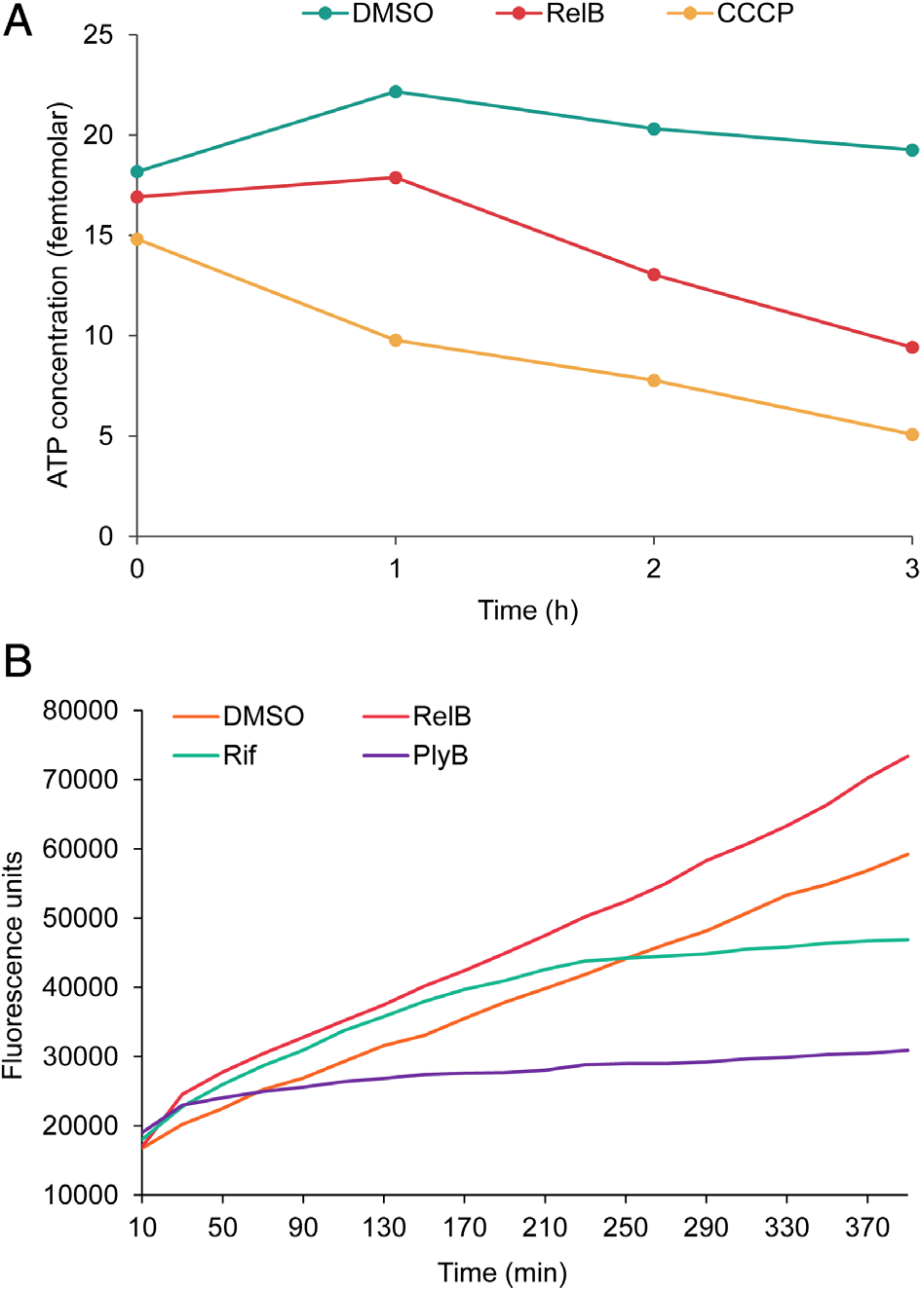
Relacidine B affects the ATP and NADH level of cells. A. ATP concentration of *X*. *campestris* pv. *campestris* cells treated with compounds. Relacidine B was added at a concentration of 0.25 μg ml^−1^. CCCP (40 μg ml^−1^) was used as a positive control and DMSO is used as a negative control. B. NADH level of *X*. *campestris* pv. *campestris* cells treated with antimicrobials. Resazurin was added to the culture at a final concentration of 0.1 mg ml^−1^. Relacidine B, rifampicin, and polymyxin B were added at final concentrations of 0.25 μg ml^−1^, 0.125 μg ml^−1^, and 1 μg ml^−1^, respectively. DMSO was used as a negative control. RelB, relacidine B; Rif, rifampicin; PlyB, polymyxin B.

Next, the effects of relacidine B on cellular metabolism were studied. The addition of relacidine B caused a drop in the intracellular ATP concentration of cells compared to DMSO‐treated cells (negative control, Fig. 3A). The positive control, carbonyl cyanide *m*‐chlorophenyl hydrazone (CCCP), a compound known to inhibit the oxidative phosphorylation of cells, caused a more drastic drop in intracellular ATP. Notably, cells treated with relacidine B for up to 2 h did not affect the growth curve (Fig. 2A) and the integrity of the cell membrane (Fig. 2), suggesting that the ATP drop is caused by the cellular metabolism but not the cell death. A possible explanation for the decrease in intracellular ATP would be that ATP synthetase is targeted by relacidine B. This is unlikely however, as *E. coli* cells, in which enhanced substrate‐level phosphorylation (Jensen and Michelsen, 1992) can compensate for the absence of ATP synthetase, are also killed by relacidine B. A comparison of the sensitivity of wild type and *atp* (ATP synthetase gene) mutants of *E. coli* to relacidine B confirmed this, showing similar MIC values for all strains (Supporting Information Table S2).

To further investigate the cause of the decrease of ATP levels in bacteria treated with relacidine B, the NADH level of cells was determined using resazurin, which is reduced to resorufin by NADH in the presence of NADH dehydrogenase (De Jong and Woodlief, 1977; Barnes and Spenney, 1980; Winartasaputra *et al*., 1980; Hanson and Freier, 1983; Shahangian *et al*., 1984). The NADH level of cells treated with relacidine B was higher than that of the control, although both of them showed the same trends of increase, whereas polymyxin B and rifampicin blocked the reduction after 30 min and 250 min respectively (Fig. 3B). This result indicates that relacidine B increases NADH of cells. The increase of the NADH level and the decrease of the ATP level suggest that the oxidative phosphorylation process of cells is affected by relacidine B (Supporting Information Fig. S7). More precisely, either the electron transport chain is disrupted or ATP synthase is uncoupled from the electron transport chain (ETC).

### Expression of relacidines during the interaction with pathogens

**Fig 4:**
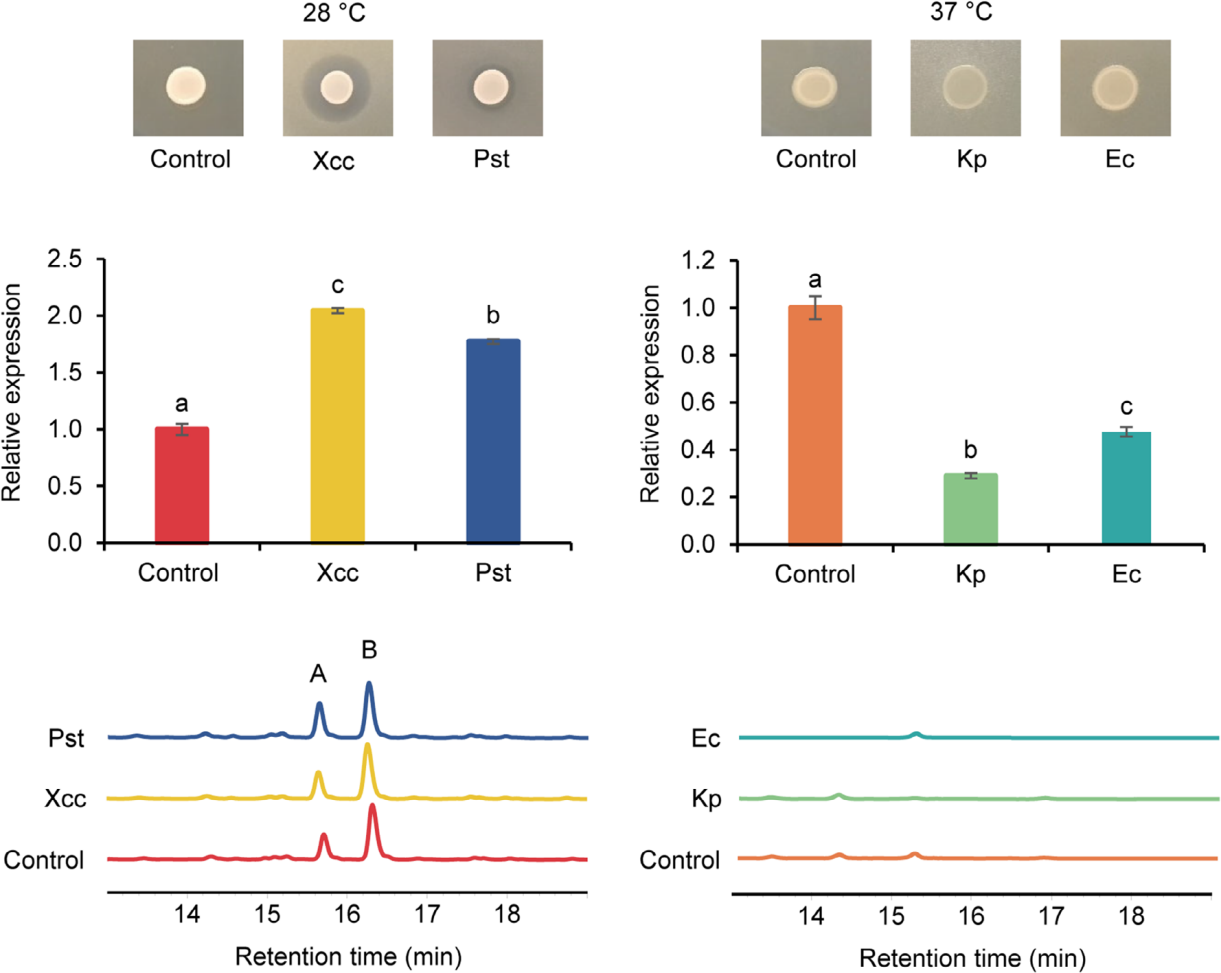
Investigation of expression of relacidines during the interaction with two plant pathogens (left panel) and two human pathogens (right panel) at transcriptional and metabolic levels. The expression of a relacidine biosynthetic gene (*rlcC*) was tested with real‐time PCR using three replicates. Different lowercase letters indicate a significant difference (ANOVA, *P* < 0.001) between treatments. The production of relacidines was analysed by HPLC. Controls are producers growing on LB plates without pathogens. Xcc, *X*. *campestris* pv. *campestris* NCCB92058; Pst, *P*. *syringae* pv. *tomato* DC3000; Kp, *K. pneumoniae* LMG20218; Ec, *E. coli* ET8. Relacidine A and relacidine B in the HPLC chromatogram are indicated as A and B respectively.

Various strains of *B. laterosporus* have been reported to have good biocontrol potential (de Oliveira *et al*., 2004; Saikia *et al*., 2011; Prasanna *et al*., 2013; Miljkovic *et al*., 2019). A *B. laterosporus* strain isolated from honeybees was even reported to have a probiotic effect on the host (Khaled *et al*., 2018). Notably, all of these biocontrol strains (if the genomic sequence is available) were found to harbour an unidentified BGC that showed high similarity to relacidines (Supporting Information Fig. S8). Given the effectiveness of relacidines in combating pathogens, the potential of their newly isolated producer, *B. laterosporus* MG64, to be used as a biocontrol agent or probiotic culture was evaluated. The expression and production of relacidines during the interaction with two plant pathogens (*X*. *campestris* pv. *campestris* and *P*. *syringae* pv. *tomato*) and two human pathogens (*K. pneumoniae* and *E. coli*) were investigated. As shown in Fig. 4, *B. laterosporus* MG64 displayed a clear inhibition activity against *X*. *campestris* pv. *campestris* and *P*. *syringae* pv. *tomato* grown at 28°C. Both plant pathogens clearly induced the expression of the core biosynthetic gene (*rlcC*) of the BGC. The relacidine peptides were also discovered in the cell extracts; however, the presence of the pathogens did not seem to result in higher amounts of relacidine in the cell extracts. No inhibition was observed when *B. laterosporus* MG64 was cocultured with *K. pneumoniae* and *E. coli* at 37°C, even though its secondary metabolites, relacidines, displayed potent activity against these two pathogens. Further inspection of the transcripts revealed that *rlcC* was expressed when growing alone, but downregulated when interacting with the two human pathogens and that no relacidine peptides were detected in the cell extracts, regardless of the presence of the human pathogens. Together, these results suggest *B. laterosporus* MG64 can be a good biocontrol strain for plants, but it is likely not suitable to be used as probiotic.

## Discussion

Here, we identified two relacidines as members of a novel class of cationic cyclic lipopeptides. They have both similarities and differences compared to brevicidine and laterocidine, which are also antimicrobials produced by *Brevibacillus laterosporus* (Li *et al*., 2018). Brevicidine is different from relacidines in the first amino acid residue (Ser‐1 in relacidines is replaced by Asn‐1 in brevicidine) and the lactone ring (the Gly‐13 in relacidines is absent in brevicidine) (Supporting Information Fig. S9). Laterocidine is different from relacidines in the fatty acid side chain and the lactone ring: C_7_H_13_O_1_ and Gly‐Ser‐Gly/Ala in relacidines are replaced by C_9_H_17_O_1_ and Asn‐Gly‐Gly in laterocidine, respectively (Supporting Information Fig. S9). Apart from the differences, brevicidine, laterocidine, and relacidines share outstanding characteristics. All of them contain a linear cationic chain and a hydrophobic lactone ring (Supporting Information Fig. S9), both of which were considered crucial for the antibacterial activity (Li *et al*., 2018).

The structure of relacidines, as well as of the recently reported brevicidine and laterocidine, is novel compared with other cationic cyclic lipopeptides such as tyrocidines, gramicidin S, and polymyxins (Supporting Information Fig. S10), some of which are considered antibiotics of the last resort for combating multidrug‐resistant bacteria (Mogi and Kita, 2009). First of all, they contain a cationic linear fragment constituted with a fatty acid tail and eight amino acid residues. Gramicidin S and tyrocidines do not possess a linear fragment and polymyxins contain a shorter cationic linear fragment constituted with a fatty acid tail and three amino acid residues (Supporting Information Fig. S10). Also, the ring of relacidines is formed by an ester bond, in contrast to the peptide bond found in gramicidin S, tyrocidines, and polymyxins (Supporting Information Fig. S10). Moreover, the ring of relacidines is constituted with five amino acid residues while that of gramicidin S, tyrocidines, and polymyxins contain 10, 10, and 7 amino acid residues, respectively (Supporting Information Fig. S10). Lastly, the ring of relacidines is uncharged, unlike gramicidin S, tyrocidines, and polymyxins have 2, 1, and 4 positive charges, respectively (Supporting Information Fig. S10).

Relacidines selectively combat Gram‐negative pathogens, which is similar to the structurally related peptides brevicidine and laterocidine (Li *et al*., 2018). Another class of cationic peptides, polymyxins, also displays potent activity against Gram‐negative bacteria but not against Gram‐positive bacteria. The similarity of the inhibitory spectra of these peptides is believed to be related to their structures. Both relacidines and polymyxins possess a cationic linear fragment, which is proven to be important for binding to the anionic LPS of Gram‐negative cells (Ntwasa, 2012). Apart from the inhibitory spectra, the antimicrobial potency of relacidines is comparable to that of polymyxins, which are used in the treatment of infections caused by multi‐drug resistant Gram‐negative bacteria. Moreover, research on brevicidine and laterocidine showed that they have a low risk of resistance development in *E. coli* (Li *et al*., 2018). This is of paramount importance since the multidrug‐resistant bacteria have become a major concern in recent decades. All these factors show the great potential of relacidines, brevicidine, and laterocidine for pharmaceutical applications.

Likely because of the structural uniqueness, relacidines, as well as brevicidine and laterocidine, display a different mode of action compared to other cationic cyclic peptides, which disrupt the cellular membranes and resulting in cell death. Our results show that relacidines bind to LPS but do not form pores in the cell membrane (Fig. 2C). This is in line with an investigation on brevicidine and laterocidine (Li *et al*., 2018). We also provide evidence to show that relacidines do not affect the biosynthesis of peptidoglycan and RNA (Supporting Information Figs. S4‐S6). Instead, they affect the oxidative phosphorylation of cells, thus disrupting the biosynthesis of ATP (Fig. 3A), which supplies energy for metabolisms. There are two possible mechanisms employed by relacidines to affect the oxidative phosphorylation. First of all, it can be an uncoupler of ATP synthase and the ETC. The ETC is a process of electron transfer from electron donors to electron acceptors. Meanwhile, it couples the transfer of protons across the cellular membrane, thus creating a proton‐motive force. An uncoupler can disrupt the proton gradient and affect the biosynthesis of ATP. CCCP is a typical uncoupler that carries protons across the membrane and disrupts the biosynthesis of ATP (Heytler *et al*., 1962; Heytler, 1963). Additionally, relacidine B can also be an inhibitor of the protein complexes that constitute the electron transport chain, thus disrupting proton export. Examples of such compounds are rotenone and antimycin A that bind to complex I and complex III, respectively (Palmer *et al*., 1968; Alexandre *et al*., 1984; Campo *et al*., 1992; Maguire *et al*., 1992; Xia *et al*., 1997; Ma *et al*., 2011). Both mechanisms result in a higher intracellular proton concentration, thus explaining the hyperpolarization of the cellular membrane (Fig. 2D). Future studies will be directed to reveal which exact mechanism is employed by relacidines.

Our previous study showed that *B. laterosporus* MG64 can inhibit a broad range of pathogens (Li *et al*., 2020). In this study, by inspecting the production of the potent antimicrobial relacidines during the interaction with plant pathogens, we show that *B. laterosporus* MG64 can be applied as a biocontrol agent. The application of *B. laterosporus* in biocontrol of pathogenic microorganisms, insects, and nematodes has been reported previously (de Oliveira *et al*., 2004; Tian *et al*., 2006; Saikia *et al*., 2011; Zhen *et al*., 2011; Prasanna *et al*., 2013; Panda *et al*., 2014). Some *B. laterosporus* strains were even used as probiotics (Sanders *et al*., 2003; Khaled *et al*., 2018). However, from the perspective of relacidines production during interaction with pathogens, *B. laterosporus* MG64 seems not suitable to be used as probiotics in animals, including humans. The abolishment of relacidine production is more likely a result of unfavourable growing temperatures (37°C) than inhibition by pathogens (Fig. 4).

## Experimental procedures

### Extraction and purification of antimicrobial compounds

The producing strain, *B. laterosporus* MG64, was grown in Lennox broth (LB) overnight. The overnight culture was then diluted 100 times in fresh LB broth and incubated at 28°C with agitation for 18 h. The supernatant was collected by centrifuging at 10 000*g* for 10 min and the secondary metabolites were precipitated with ammonium sulfate to 40% saturation. The precipitates were then dissolved in Milli‐Q water and filtered through a 0.45 μm cellulose acetate membrane to achieve crude extracts. The crude extracts were applied to a reverse high‐performance liquid chromatography (HPLC) for purification. An analytical C‐18 column was used and the mobile phases were HPLC‐grade water supplemented with 0.1% trifluoroacetic acid (TFA) (solvent A) and acetonitrile supplemented with 0.1% TFA (solvent B). The compounds were eluted with a linear gradient of solvent B (from 15% to 45%) in 30 min at a flow rate of 1.0 ml min^−1^. A UV‐detector set at a wavelength of 280 nm was used to monitor the effluents. All the peaks were collected separately and their antibacterial activities were tested against a plant pathogen *Xanthomonas campestris* pv. *campestris*.

### LC–MS/MS analysis

The active compounds were characterized by liquid chromatography–tandem mass spectrometry (LC–MS/MS). An Ultimate 3000 UHPLC system coupled with a Q‐Exactive Orbitrap™‐based mass spectrometer (Thermo Scientific, San Jose, CA, USA) was used. The UHPLC system was equipped with a kinetex WVO‐C18 column (2.6 μm particles, 100 × 2.1 mm, Phenomenex) and the mass spectrometer was equipped with a HESI‐II electrospray source. The mobile phases of the LC were water with 0.1% formic acid and acetonitrile with 0.1% formic acid. In each run, a 10 μl sample was injected and separated with mobile phases at a flow rate of 500 μl min^−1^. The MS/MS data were acquired with a spray voltage of 3.5 kV (positive mode) and a capillary temperature of 275°C. The m/z range was set to 300–2000 and the resolution was 70 000. MS/MS data were recorded in targeted MSMS using PRM mode. In order to investigate the amino acid composition of the lactone ring, peptides were hydrolyzed in 2 M NaOH and desalted with an open column filled with C18 silica gel spherical (Sigma, USA) before applying to LC–MS/MS.

### NMR spectroscopy

Prior to NMR analysis, the isolated peptide was purified further by RP‐HPLC (XBridge C8 250 mm analytical column, solvent A: 0.1% FA in ACN, solvent B: 0.1% FA in ddH_2_O, gradient 90% B to 30% B over 40 min, flow: 0.5 ml min^−1^) and the pure fractions were pooled and lyophilized. Around 1 mg of compound was dissolved in 0.5 ml DMSO‐*d*_6_. A Brüker Ascend 600 MHz spectrometer was used to record ^1^H NMR, ^1^H‐^1^H‐COSY NMR, ^1^H‐^1^H‐TOCSY NMR, and ^1^H‐^1^H‐NOESY NMR spectra. Chemical shifts in ^1^H NMR spectra were internally referenced to solvent signals (DMSO‐*d*_*6*_ at δH = 2.50 ppm, δC = 39.51 ppm).

### Minimal inhibitory concentration test

The mnimal inhibitory concentrations (MICs) of relacidine A and B were tested with the standard broth dilution method (Wiegand *et al*., 2008). Polymyxin B was used as a reference. MHB medium was used for all the bacteria tested. Peptides were diluted from 32 μg ml^−1^ to 0.06 μg ml^−1^ in a twofold serial dilution and cells were adjusted to a concentration of 5.0 × 10^5^ CFU ml^−1^. The 96‐well plate was incubated at 28°C for 36 h and the OD_600_ was measured with a Tecan Infinite F200 Pro Luminometer. The lowest concentration that causes invisible growth of bacteria was defined as the MIC values. The experiment was done in quadruplicate for each peptide and each strain.

### Bacterial growth curve and time‐kill assay

*X. campestris* pv. *campestris* was first inoculated in LB broth and incubated at 28°C overnight. The overnight culture was then diluted with fresh LB to an OD_600_ of 0.03 and distributed in a 96‐well plate. The plate was incubated in a microplate spectrophotometer (1000 rpm, 28°C) until OD_600_ reached 0.05. Relacidines was added at concentrations of 0.25 μg ml^−1^ (1 × MIC) and 2.5 μg ml^−1^ (10 × MIC). Polymyxin B was added at a concentration of 0.625 μg ml^−1^ (10 × MIC, lower concentration of polymyxin B does not affect the growth of cells although its MIC value is ≤0.06 μg ml^−1^). The same amount of DMSO was added as a control. The plate was incubated at the same condition for 20 h and the growth kinetics were monitored. Each treatment was done in quadruplicate. For the time‐kill assay, all compounds were added at a concentration of 2.5 μg ml^−1^ when the OD_600_ reached 0.1. At each time point (0, 30, 60, and 90 min), 10 μl of culture was tenfold serially diluted and plated on LB agar plates. After incubation at 28°C for 36 h, colonies were counted and the number of CFU per mL was calculated.

### Membrane permeability assay

A commercial LIVE/DEAD Baclight Bacterial Viability Kit (Invitrogen) was used to test the integrity of the cell membrane of *X. campestris* pv. *campestris* after treatment with peptides. Cells were grown in LB overnight and diluted to an OD_600_ of 0.2. Relacidine B was added at a concentration of 0.25 μg ml^−1^ (1 × MIC), while polymyxin B was added at a concentration of 1 μg ml^−1^. The same amount of DMSO was added to the control. Cells were treated at room temperature for 30 min before harvest. The harvested cells were washed and resuspended in 200 μl 0.9% saline (NaCl) solution. Two different dyes (3.34 mM SYTO9 and 20 mM propidium iodide) were added at a ratio of 1:1 (v/v). Cells were stained in the dark for 15 min and a 5 μl sample was mounted on a 1% agarose pad before being imaged using a Nikon Ti‐E microscope (Nikon Instruments, Tokyo, Japan) equipped with a Hamamatsu Orca Flash 4.0 camera.

### Membrane potential assay

Fresh *X. campestris* pv. *campestris* cells were inoculated in MHB medium and grown until OD_600_ reached 0.4. The culture was two times diluted with fresh MHB medium and dispensed in a 96 well plate. The membrane potential dye DiSC(3)‐5 was added to a final concentration of 6 μM. The fluorescence signal (excitation 633 nm, emission 660 nm) was allowed to stabilize for 20 min in a microplate spectrophotometer (BioTek Synergy Mx). After stabilization, relacidine B and polymyxin B were added at final concentrations of 0.5 μg ml^−1^ and 1 μg ml^−^ respectively. Milli‐Q water was used as a negative control. Blanks (without cells) were used to show that the signal change is not caused by the interaction of the dye and compounds. The fluorescence signal was then monitored for 60 min at an interval of 5 min with a microplate spectrophotometer. Three replicates were used for each treatment.

### HADA incorporation assay

d‐amino acids are involved in peptidoglycan biosynthesis of diverse bacteria (Lam *et al*., 2009). HADA is a fluorescent d‐amino acid analogue that can be used for monitoring the peptidoglycan synthesis activity of bacteria (Kuru *et al*., 2015). Cells were grown in LB broth until OD_600_ reached 0.2. Relacidine B was added at a concentration of 0.25 μg ml^−1^ and cells were kept growing for another 2, 4, and 5 h. DMSO was used as a negative control while ceftriaxone (4 μg ml^−1^) was used as a positive control. At each time point, cells were treated with 500 μM HADA for 24 min. After treatment, cells were washed twice with PBS buffer. Incorporation of HADA was inspected with a Nikon Ti‐E microscope (Tokyo, Japan) equipped with a Hamamatsu Orca Flash 4.0 camera.

### Lipid II binding assay

Generally, lipid II from Gram‐negative bacteria has *meso*‐diaminopimelic acid (mDAP) on residue 3, while that from Gram‐positive bacteria has lysine (Cochrane *et al*., 2016). Both types were used in the lipid II binding assay. An overnight culture of the indicator strain (*X. campestris* pv. *campestris*) was added to melted LB agar at a final concentration of 0.2% (v/v) before pouring plates. Lipid II and relacidine B were mixed at different ratios (0.5, 1, and 2) before spotting onto the plate. The plate was incubated at 28°C overnight. The binding of lipid II and relacidine B is indicated by the decrease of halo size.

### Intracellular ATP concentration assay

The BacTiter‐Glo™ Microbial cell viability assay kit (Promega) was used to determine the intracellular ATP concentration of *X. campestris* pv. *campestris*. Cells were grown in LB broth until OD_600_ reached 0.2. Relacidine B was added at a concentration of 0.25 μg ml^−1^ (1 × MIC) and the DMSO was used as a solvent control. Carbonyl cyanide *m*‐chlorophenyl hydrazine (CCCP), an uncoupler of oxidative phosphorylation, was used as a positive control (40 μg ml^−1^). Cells were treated at 28°C for 0, 1, 2, and 3 h. At each time point, 100 μl of cell culture was added to 100 μl BacTiter‐Glo™ reagent and incubated at room temperature for 5 min. Luminescence was with a Tecan Infinite F200 Pro luminometer and the intracellular ATP concentration was calculated with a standard curve made with a commercial ATP solution.

### Resazurin assay

Nonfluorescent resazurin can be reduced to fluorescent resorufin in a NAD(P)H dependent manner in the presence of NAD(P)H dehydrogenase (De Jong and Woodlief, 1977; Barnes and Spenney, 1980; Winartasaputra *et al*., 1980; Hanson and Freier, 1983; Shahangian *et al*., 1984). Therefore, the resazurin/NAD(P)H dehydrogenase/NAD(P)H system can be used to detect the NAD(P)H level of cells. *X. campestris* pv. *campestris* cells were grown in LB broth until OD_600_ reached 0.1. Resazurin was added to the culture at a final concentration of 0.1 mg ml^−1^. Relacidine B, rifampicin, and polymyxin B were added at final concentrations of 0.25, 0.125, and 1 μg ml^−1^ respectively. DMSO was used as a negative control. Blanks (without cells) revealed that the signal change is not caused by the interaction of resazurin and compounds. Fluorescence was recorded at a wavelength of 560 nm every 20 min over a period of 4 h with a BioTek Synergy Mx 96‐well plate reader.

### Determining relacidine expression during the interaction with different pathogens

A fresh culture of each pathogen was mixed with melted agar (around 45°C) before preparing plates. *B. laterosporus* MG64 was inoculated (10 μl inoculum with an OD_600_ of 1.0) at the centre of each plate containing pathogens. The plates were incubated at 28°C or 37°C based on the growth temperature of the indicator strain. After 2 days, the inhibition against pathogens was documented with photos and *B. laterosporus* MG64 cells were collected for further analysis. The total RNA was extracted using the High Pure RNA isolation kit (Roche Diagnostics Nederland BV) and 500 μg of each RNA sample was used for reverse transcription using the SuperScript™ III Reverse Transcriptase (Invitrogen) following the instruction manual. The cDNAs were five times diluted to make templates. The primers used in the qPCR are as follows: Housekeeping gene: *rpoB*‐forward, CCAAGACATTTCGCCAATCC; *rpoB*‐reverse, CGTTCCTTCGACTCGTCTAC. Target gene: *rlcC*‐forward, TCGACAGTACGATGCCTTTC; *rlcC*‐reverse, GAGCTTCGCCATCAACAC. The qPCR mixture contained with 1 μl template, 0.6 μl forward primer, 0.6 μl reverse primer, 7.8 μl distilled water, and 10 μl SsoAdvanced™ Universal SYBR Green Supermix (Bio‐Rad). The procedure is 95°C 3 min, 95°C 30 s, 57°C 30 s, 72°C 30 s, 72°C 5 min and 40 cycles were used. The relative expression of *rlcC* is calculated with the 2^‐ΔΔCT^ method. The metabolites were extracted from the cells with 50% acetonitrile and lyophilized. The compounds were redissolved in Milli‐Q water and filtered through a 0.45 μm cellulose acetate membrane before applying to HPLC and MALDI‐TOF analysis.

## Supporting information


**Appendix S1:** Supporting information
