# Supplementary material for: Characterization of two relacidines belonging to a novel class of circular lipopeptides that act against Gram‐negative bacterial pathogens
Source: Environ Microbiol. 2020 Jul 20;22(12):5125–36. doi: 10.1111/1462-2920.15145 (PMC7818431; doi:10.1111/1462-2920.15145)
Supplement: Supplementary file 1 — Appendix S1: Supporting information [file EMI-22-5125-s001.docx]

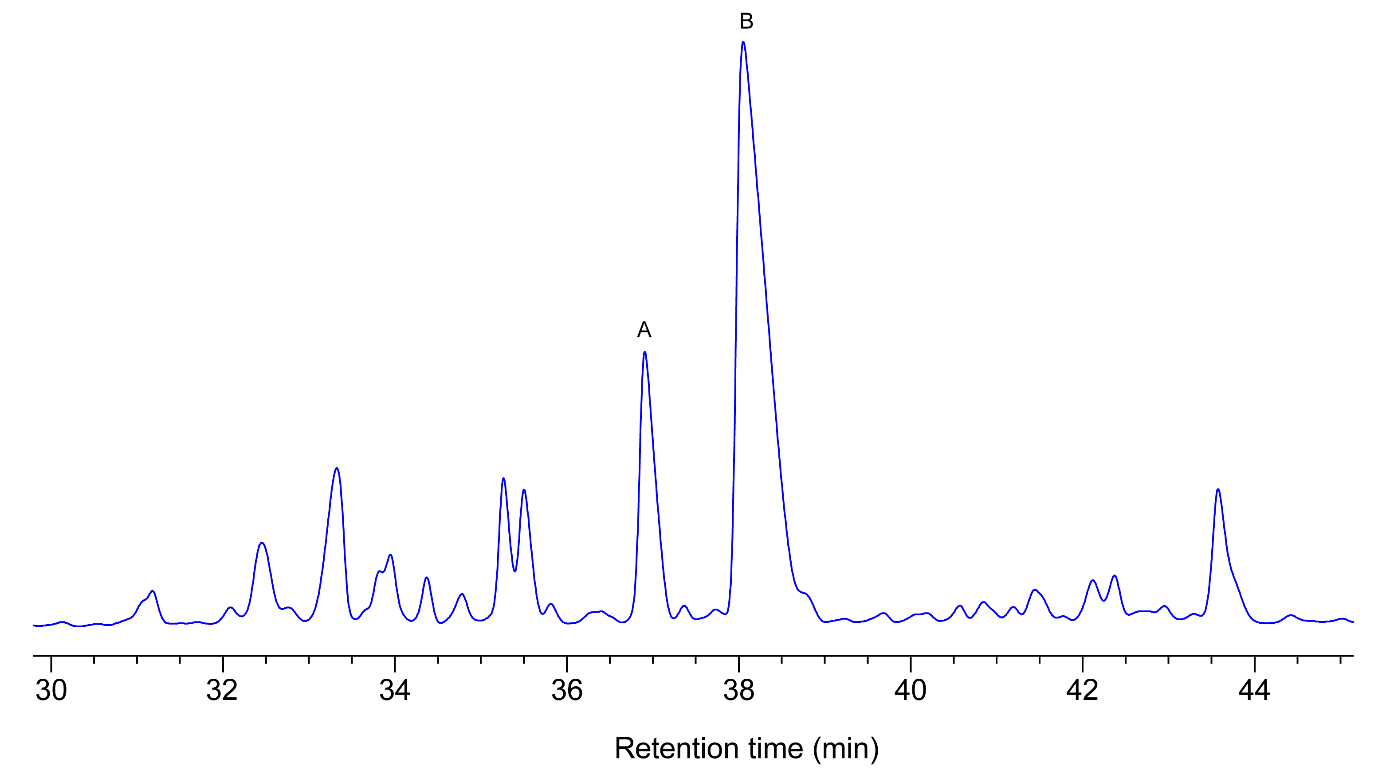


Figure S1. HPLC chromatogram of bioactive compounds extracted from *B. laterosporus* MG64. The two peaks showed the most potent activity toward *X. campestris* pv. *campestris* were labeled as A and B, respectively.


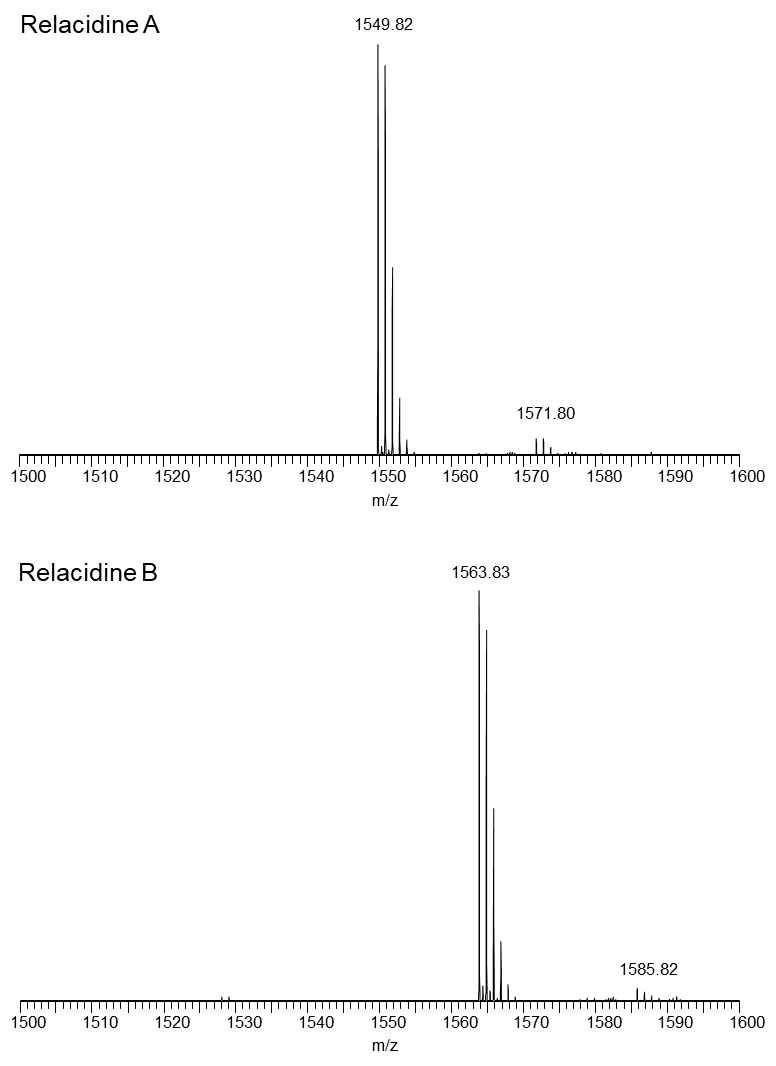


Figure S2. LC-MS analysis of the two bioactive compounds isolated from *B. laterosporus* MG64. The ions at *m/z* (z = 1) 1549.82 and 1563.83 represent the singly protonated [M + H]^+^ of relacidine A-B, respectively. Their sodium-cationized [M + Na]^+^ are 1571.80 and 1585.82, respectively.


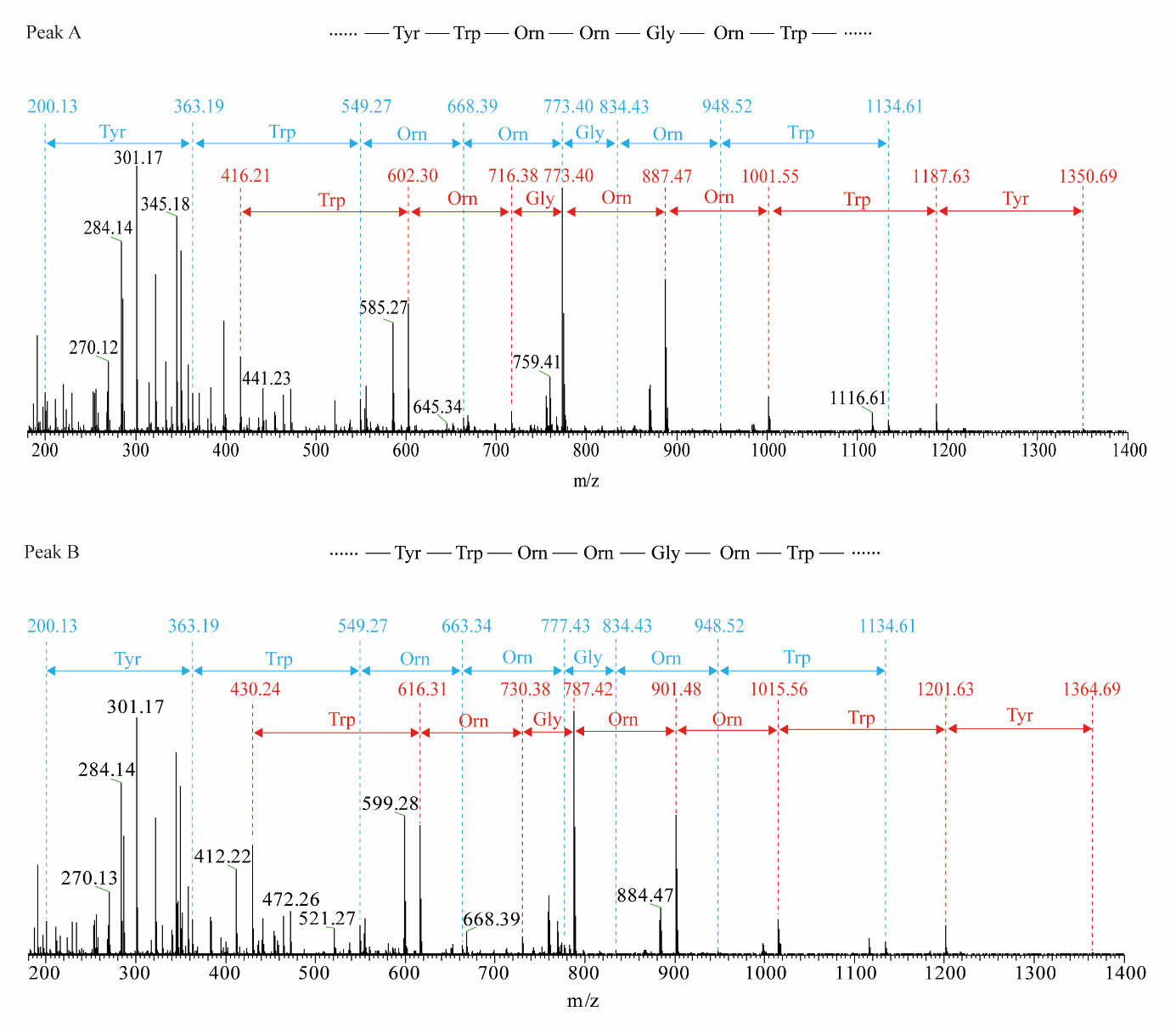


Figure S3. Fragmentation of relacidines by tandem MS to generate *b* and *y* ions. *b* ions are indicated in blue while *y* ions are indicated in red. (A) Fragmentation of relacidine A. (B) Fragmentation of relacidine B.

Table S1. Chemical shift (ppm) assignments of relacidine B (*d_6_*-DMSO).

| **Amino acid** | **Position** | **δ ^13^C NMR** | **δ ^1^H NMR** | **Amino acid** | **Position** | **δ ^13^C NMR** | **δ ^1^H NMR** |
| --- | --- | --- | --- | --- | --- | --- | --- |
| **Ser1** | 1-NH |  | 7.95 | **Trp8** | 28-NH |  | 8.28 |
|  | 1 | 54.6 | 4.29 |  | 28 | 52.9 | 4.68 |
|  | 2 | 61.4 | 3.50 |  | 29 | 27.1 | 3.11 / 2.96 |
| **Tyr2** | 3-NH |  | 8.04 |  | 30 | 123.8 | 7.11 |
|  | 3 | 54.5 | 4.32 |  | 31 |  | 10.84 |
|  | 4 | 35.9 | 2.81 / 2.64 |  | 32 | 111.0 | 7.31 |
|  | 5 | 129.8 | 6.88 |  | 33 | 120.6 | 7.04 |
|  | 6 | 114.6 | 6.56 |  | 34 | 118.0 | 6.96 |
| **Trp3** | 7-NH |  | 8.14 |  | 35 | 118.2 | 7.57 |
|  | 7 | 53.3 | 4.51 | **Thr9** | 36-NH |  | 7.98 |
|  | 8 | 27.1 | 3.14 / 2.93 |  | 36 | 53.2 | 4.64 |
|  | 9 | 123.6 | 7.14 |  | 37 | 69.4 | 5.00 |
|  | 10 |  | 10.81 |  | 38 | 14.1 | 0.98 |
|  | 11 | 111.0 | 7.31 | **Ile10** | 39-NH |  | 7.73 |
|  | 12 | 120.6 | 7.04 |  | 39 | 56.5 | 4.15 |
|  | 13 | 118.0 | 6.96 |  | 40 | 35.4 | 1.64 |
|  | 14 | 118.2 | 7.57 |  | 41 | 24.7 | 1.53 / 1.10 |
| **Orn4** | 15-NH |  | 8.04 |  | 42 | 10.5 | 0.86 |
|  | 15 | 51.5 | 4.32 |  | 43 | 14.8 | 0.85 |
|  | 16 | 29.0 | 1.72 / 1.57 | **Gly11** | 44-NH |  | 9.20 |
|  | 17 | 24.4 | 1.51 |  | 44 | 43.5 | 3.90 / 3.35 |
|  | 18 | 38.6 | 2.73 | **Ser12** | 45-NH |  | 8.47 |
| **Orn5** | 19-NH |  | 8.23 |  | 45 | 56.3 | 4.10 |
|  | 19 | 51.6 | 4.34 |  | 46 | 60.4 | 3.80 / 3.68 |
|  | 20 | 29.0 | 1.72 / 1.58 | **Ala13** | 47-NH |  | 7.74 |
|  | 21 | 24.4 | 1.50 |  | 47 | 48.0 | 4.39 |
|  | 22 | 38.6 | 2.69 |  | 48 | 16.9 | 1.41 |
| **Gly6** | 23-NH |  | 8.32 | **FA** | F-1 | 32.6 | 2.11 |
|  | 23 | 41.7 | 3.77 / 3.72 |  | F-2 | 31.5 | 1.50 / 1.27 |
| **Orn7** | 24-NH |  | 8.05 |  | F-3 | 33.3 | 1.26 |
|  | 24 | 51.5 | 4.32 |  | F-4 | 28.4 | 1.28 / 1.09 |
|  | 25 | 29.2 | 1.42 / 1.29 |  | F-5 | 10.9 | 0.81 |
|  | 26 | 24.4 | 1.27 |  | F-6 | 18.6 | 0.81 |
|  | 27 | overlap | 2.51 |  |  |  |  |

**1HNMR of relacidine B (*d_6_*-DMSO)**

^1^H-^1^H-TOCSY NMR of relacidine B (*d_6_*-DMSO)

^1^H-^1^H-COSY-NMR of relacidine B (*d_6_*-DMSO)

^13^C-^1^H-HSQC NMR of relacidine B (*d_6_*-DMSO)

^1^H-^1^H-NOESY NMR of relacidine B (*d_6_*-DMSO)

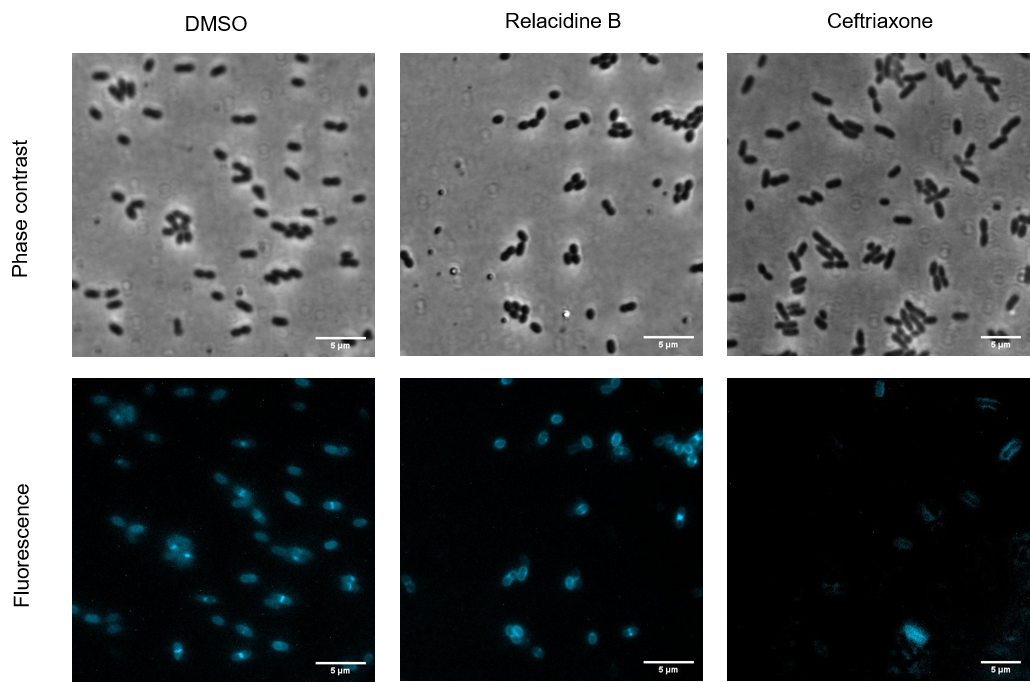


Figure S4. The effects of peptides on HADA incorporation during cell wall biosynthesis. DMSO was used as a negative control and ceftriaxone (4 µg/mL) was used as a positive control. *X*. *campestris* pv. *campestris* cells were treated with compounds (up to 5 h for DMSO and relacidine B and 30 min for ceftriaxone) and pulsed with HADA for 30 min. The white bar represents 5 µm. The signal indicates the incorporation of HADA, which is abundantly present in DMSO and relacidine B treatments and barely present in ceftriaxone treatment.


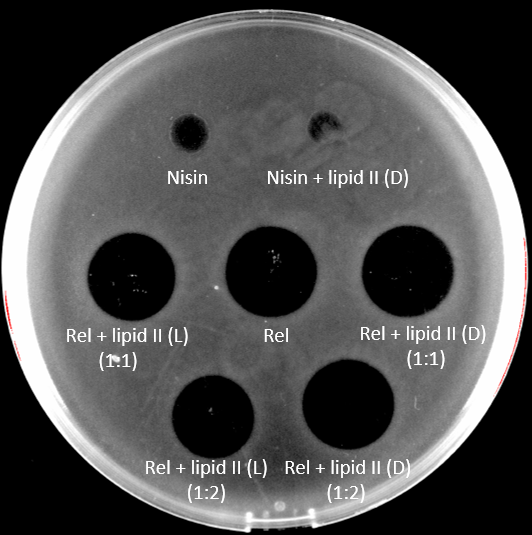


Figure S5. Lipid II was mixed with compounds at different ratios before spotting onto the plate, which contains the indicator strain *X. campestris* pv. *campestris*. Nisin was used as a negative control. D or L in the bracket means a Gram-negative or Gram-positive type of lipid II was used. The plate was incubated at 28 °C overnight before recording the result.


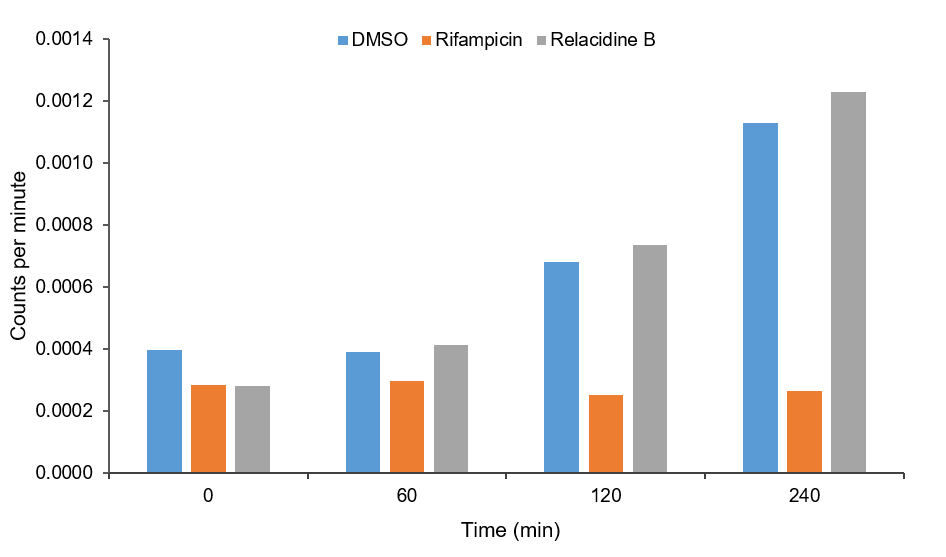


Figure S6. RNA biosynthesis of *X. campestris* pv. *campestris* after treating with compounds. [5^3^H] uridine was used as a precursor for RNA biosynthesis. Radioactivity was recorded by liquid scintillation counting. DMSO is a negative control while rifampicin is a positive control. Both rifampicin and relacidine B were added at a final concentration of 2×MIC. Two replicates were used for each treatment and the mean values are shown.

Table S2. MICs relacidine B against *E. coli* MG1655 wild type strain and its *atp* knock out mutants.

| Strain | MIC (µg/mL) | Strain | MIC (µg/mL) |
| --- | --- | --- | --- |
| E. coli MG1655 (WT) | 0.5 | *ΔatpE* | 1.0 |
| *ΔatpA* | 0.5 | *ΔatpF* | 1.0 |
| *ΔatpB* | 0.5 | *ΔatpG* | 1.0 |
| *ΔatpC* | 1.0 | *ΔatpH* | 0.5 |
| *ΔatpD* | 1.0 | *ΔatpI* | 1.0 |

Note: *atpA-H* are ATP synthetase biosynthetic genes.


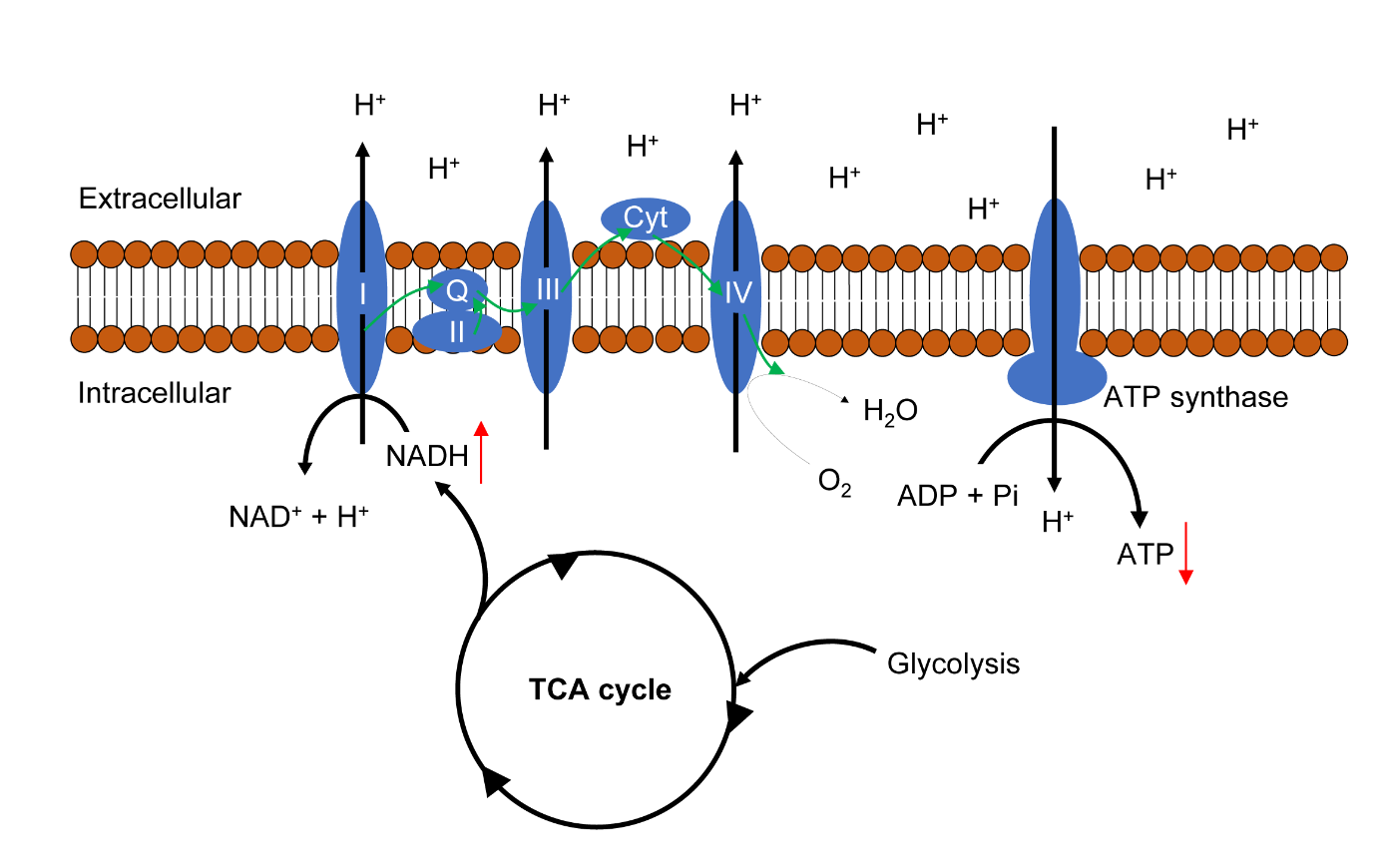


Figure S7. Schematic presentation of the effect of relacidine B on the oxidative phosphorylation of cells. Complex I, NADH dehydrogenase; complex II, succinate dehydrogenase; complex III, cytochrome c reductase; complex IV, cytochrome c oxidase; Q, quinone. The electron transport chain is indicated by a green curve. The change of NADH and ATP concentration after treatment with relacidine B are indicated with yellow arrows (up, increase; down, decrease).


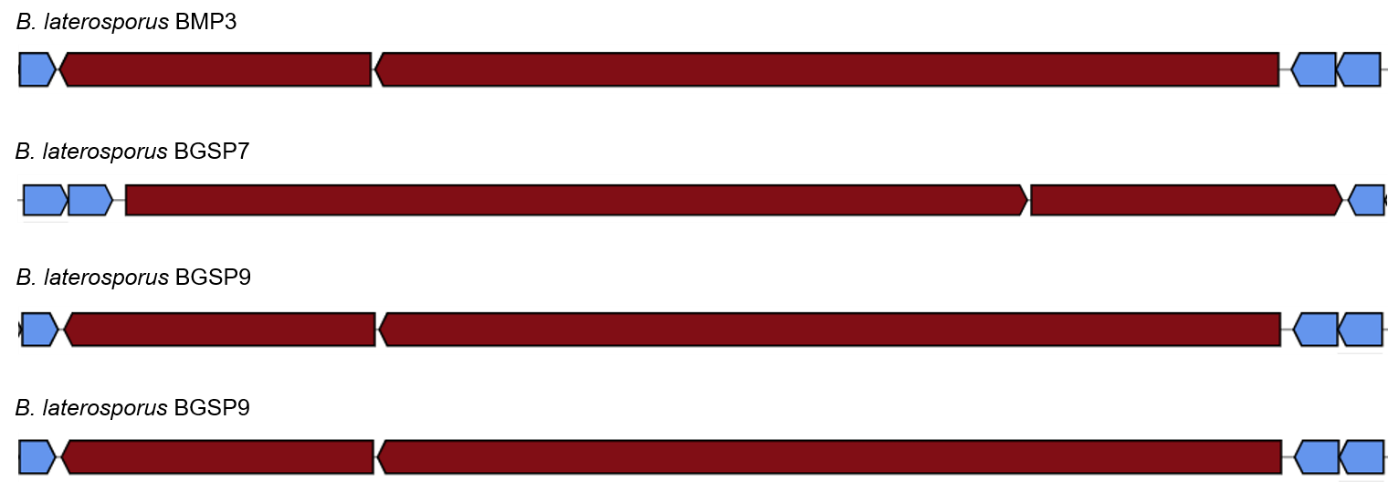


Figure S8. BGCs harbored by biocontrol strains of *B. laterosporus* that show high similarity to the relacidines BGC. Genomic sequences of the biocontrol stains were analyzed with antiSMASH.

Figure S9. Structure comparison of relacidines, brevicidine, and laterocidine. The parts of brevicidine and laterocidine that are different from relacidines were indicated in red. The thirteenth amino acid residues in relacidines were indicated in blue.


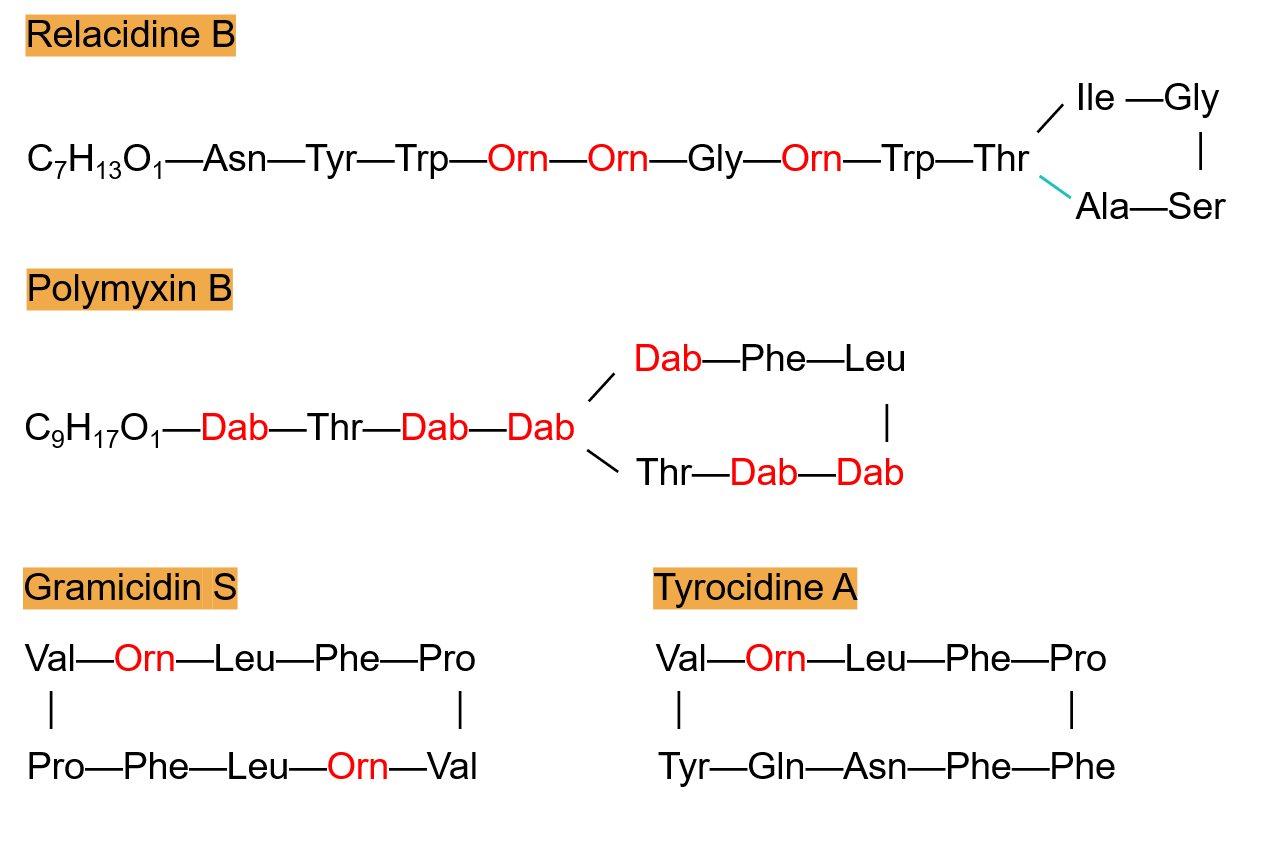


Figure S10. Structure comparison of relacidines, polymyxins, gramicidin S, and tyrocidines. The positive-charged residues are indicated in red. The ester bond in relacidine B is indicated in green.
